# Annotations

**Published:** 1905-10-28

**Authors:** 


### ANNOTATIONS.

### Dr. Barnardo's Successor.

We are rather surprised that Dr. Barnardo's successor, who was selected on Tuesday at a special meeting of members of the Association, in whose hands the control of the institution is vested, is not a medical man. In all other respects the choice of Mr. William Baker, M.A., LL.D., Chairman of the Council, is all that could be desired. Mr. Baker has been connected with the work for nearly twenty years, was Vice-Chairman of the Council for some time before he became Chairman, was not only a close personal friend of Dr. Barnardo, but throughout the whole of the period since he identified himself with the cause, was entirely in harmony with the aims and methods of the founder. Moreover, in order to undertake the extremely onerous duties of director, he has, it is stated, resigned a lucrative Chancery practice, and, as his new appointment is to be honorary, he has therefore made a sacrifice which cannot fail to create an impression upon the public mind. His legal experience may be useful, and we have no doubt whatever that he will throw himself with energy into the great task that lies before him of seeing that the Homes do not suffer more than an irreparable personal loss by the death of Dr. Barnardo. But we always thought that some measure of the success achieved by Dr. Barnardo was due to the fact that he was a fully qualified medical man, and therefore able to bring professional knowledge to bear upon the questions of health and sanitation which must so often inevitably arise. It is true that, so far as the Hospital for Sick Waifs is concerned, there is a medical officer in charge, but in respect to its administration, and that of the Homes, the late Dr. Barnardo must frequently have found that his training and experience were of considerable assistance to him, both in the task of suggestion and of supervision.

### Alcohol and the Public Conscience.

Sir Wilfrid Lawson, in delivering the sixth Lees and Raper Memorial Lecture at Birmingham on Monday, strenuously urged the application of legislative prohibition to the liquor traffic. His argument is simple enough; it is this: law should prohibit great public evils; the liquor traffic is a public evil of enormous proportions; therefore law should prohibit it. Sir Wilfrid Lawson claims to be a logician, and he challenges the keenest of his opponents to point out a flaw in this syllogism. Without any particular wish to be regarded as opponents to Sir Wilfrid Lawson, we might ask him whether he considers an earthquake to be a public evil, and, if so, whether he, as a logician, is prepared to apply his syllogism to earthquakes. The truth is, prohibitive legislation is not nearly so potent a remedy as some enthusiasts would have us believe, and it is of little use attempting to force it upon a public whose indifference to hygiene is the reproach of the twentieth century. That education in public health is sorely needed even among the leaders of men is exemplified in another part of Sir Wilfrid Lawson's address, where he refers to our milk supplies as innocent and beneficial. It is almost certain that these same milk supplies directly cause more preventable deaths than all the alcoholic beverages together. It is true, perhaps, that they do not lead to so much misery and degradation, but they are very far from being harmless. The fact is, we do not want complicated and tiresome machinery for the purpose of enforcing prohibition on an unwilling public. Those whose ardour for reform is strong enough to urge them beyond the realms of armchair philosophy will do well to devote their energies to the sanitary education of their fellow-men. The real need is for a public health conscience, which will cause people to regard the protection of their own health and that of their neighbours as an essential duty, and to refuse of their own free will to tolerate the distribution of any article of consumption which has the power to jeopardise their personal well-being.

### The Poplar Labour Colony.

Now that concerted action with regard to placing the unemployed on the land is to be taken, it is interesting to observe the results of Poplar's experiment in the same direction. We are from the first confronted with the fact that those with whom Poplar had to deal were not too well inclined to country life and work. The Union sent fifty-six men to the Salvation Army Colony at Hadleigh, and of these more than half left without notice. Of the rest, eleven remained chargeable to the colony and four left to go to work, two left to complete their militia training, one was discharged for drinking, and one was sent back to the workhouse as unsuitable. This does not look promising. On the most charitable construction, we must admit that the majority of the people who went to Hadleigh did not show much power of becoming self-supporting workers, and the only question is whether they were unable to do better, or merely did not care to do so. The fact is that, from the point of view of the pauper, the labour colony is altogether satisfactory. But before consenting to subsidise such colonies on a larger scale, most people will want to know if this period of good food, healthy work, and kind treatment has so far improved the men that they are likely to cease to be paupers in the future. For the answer to this question we must wait until the end of the present winter. If none or only a few of those who went there last year return, we shall say that the colony has served to lift them out of the pauper class. If, on the other hand, we find many of last year's colonists return—and we hope the Poplar Union will give the public full information on this point—we shall come to the conclusion that the man who has not energy to earn his own living regards life at the colony as "a soft job."

